# Test of an Electronic Program to Query Clinicians About Nonspecific Causes Reported for Pneumonia Deaths, New York City, 2012

**DOI:** 10.5888/pcd11.140282

**Published:** 2014-11-26

**Authors:** Laura Korin, Tara Das, Ann Madsen, Antonio Soto, Elizabeth Begier

**Affiliations:** Author Affiliations: Laura Korin, Public Health/Preventive Medicine Residency Program, New York City Department of Health and Mental Hygiene, New York, New York; Tara Das, Ann Madsen, Antonio Soto, Elizabeth Begier, Bureau of Vital Statistics, New York City Department of Health and Mental Hygiene, New York, New York

## Abstract

We tested an electronic cause-of-death query system at a hospital in New York City to evaluate clinicians’ reporting of cause of death. We used the system to query clinicians about all deaths assigned International Classification of Disease code J189 (pneumonia, unspecified) as the underlying cause of death. Of 29 death certificates that generated queries, 28 were updated with additional information, which led to revisions in the underlying cause of 27 deaths. The electronic system for querying reported cause of death was feasible and enabled quicker than usual responses; however, follow-up with clinicians to ensure timely, accurate, and complete responses was labor-intensive. Educating clinicians and enforcing reporting standards would reduce the time and effort required to ensure accurate and timely cause-of-death reporting.

## Objective

Cause-of-death reporting enables health departments determine program needs and effectiveness. Because clinicians often report cause of death incompletely or inaccurately, cardiovascular disease and pneumonia have been over-reported in some regions (1–5). The National Center for Health Statistics (NCHS) recommends that health departments query clinicians after death registration to obtain more detailed and accurate information (6,7). New York City’s Department of Health and Mental Hygiene (DOHMH) pilot-tested an electronic system for querying clinicians about cause of death and asking for more information when unspecified pneumonia, which is usually precipitated by a chronic condition, was reported as the underlying cause.

## Methods

We selected a 519-bed acute tertiary-care facility that reported substantially more unspecified pneumonia deaths than other New York City hospitals. In 2010, 16% of deaths at this hospital were reported as due to pneumonia or influenza, compared with 6.8% citywide.

In New York City, 93% of 2012 deaths were entirely reported through the Electronic Vital Events Registration System (EVERS) (8). In EVERS, the text provided by certifying clinicians on each death certificate is used to determine the underlying cause of death and to assign the corresponding International Classification of Disease (ICD-10) code (9). A standardized international algorithm is used to apply these ICD-10 codes either automatically by NCHS-provided software or manually by our nosologist if automated coding fails or is unavailable (10). 

Beginning March 1, 2012, we queried all deaths registered from January 1 through July 31, 2012, that were coded J189 (pneumonia, unspecified) as the underlying cause of death. Our DOHMH nosologist sent an email and system message via EVERS to certifying clinicians and copied the hospital administrative staff member responsible for death registration (Box). The message asked clinicians to submit electronically, through EVERS, either an amendment with a revised underlying cause of death or a comment within the death record stating that additional information was unavailable.

**E-mail and EVERS System Message Sent to Certifying Clinicians For All Deaths Registered From January 1 Through July 31, 2012, at a New York City Hospital That Were Assigned an ICD-10 Code of J189 (Pneumonia, Unspecified) as Underlying Cause Of Death**

Dear Doctor,

We are contacting you to obtain additional information about the cause of death that you certiﬁed for the patient with the following EVERS case ID number:

You are required by New York City Health Code, §205.03, subdivision (h) to respond within 5 days. Your response is essential in improving the quality of cause of death data. It enables the Health Department to evaluate the leading causes of death and magnitude of certain health problems within our communities. Pneumonia rarely causes death in the absence of other medical conditions. Please revisit this patient's medical record and ask yourself:

What conditions put the patient at risk for pneumonia? Please provide detailed information about the pneumonia, including organism, if possible.

Example:

Part I:

a. Left upper lobe Klebsiella pneumonia

b. Chronic obstructive pulmonary disease

c. Smoking

Part II: Coronary artery disease, diabetes mellitus

The cause of death need not be conﬁrmed but should reﬂect your best medical opinion.

Instructions for completing the cause of death section and submitting an amendment can be found by copying and pasting http://www.nyc.gov/htmI/doh/downloads/pdf/vs/inst-causeofdeath-submission.pdf into your web browser. If there were no underlying medical conditions or no further details on the pneumonia, please insert a comment into the patient's record.

Thank you for your attention and prompt reply. Questions? Call us at (212)788-4575, Monday through Friday, 8am–6pm, or email us at vitalqi@health.nyc.gov.

Sincerely,

New York City Bureau of Vital Statistics

Abbreviation: EVERS, Electronic Vital Events Registration System; ICD-10, International Classification of Disease, 10th revision (9).

The New York City health code mandates responses to DOHMH information requests within 5 business days (11). Nonresponses were followed with an email and an EVERS message after 2 weeks and with a telephone call after 4 weeks.

DOHMH quality assurance staff reviewed clinician amendments and comments. Our nosologists applied ICD-10 coding to approved amendments by using the same methods used during death registration. For rejected amendments, quality assurance staff or DOHMH physicians called the clinician to discuss the case.

A DOHMH physician trained in cause-of-death documentation who was temporarily assigned to the Bureau of Vital Statistics reviewed medical records of queried cases to determine the correct cause of death. The nosologist then manually assigned codes to the corrected underlying causes for comparison with original and amended causes reported.

## Results

Of the 29 death certificates we queried (4.6% of the 633 death certificates registered by the hospital from January 1 through July 31, 2012), 12 (41.3%) required a second message because of nonresponse. Clinicians responded to 22 queries (75.9% of all queries) by August 31, 2012, which was the end of the 4-week follow-up period for the last query sent. After further outreach to administrators, another 6 query responses were received by October 16, 2012, for a final response rate of 96.6%. All amendments were reviewed and accepted electronically.

Of the 28 amended cases, 27 (96.4%) amendments led to a change in the underlying ICD-10 code to one other than J189 (Table). Twelve (42.9%) amendments were inadequate in that they provided only more specific information about the infecting organism rather than providing information about the medical condition(s) that made the patient vulnerable to acquiring and dying of pneumonia.

**Table:** Post-Query Amended Cause of Death and Assigned ICD-10 Code^a^ Compared With Results of DOHMH Physician Chart Review, Test of an Electronic Program to Query Clinicians About Nonspecific Causes Reported in Pneumonia Deaths, New York City, March 1–October 16, 2012

Amended Cause of Death	Amended ICD-10 Code	Cause of Death, Physician Review	ICD-10 Code, Physician Review
Amended underlying cause of death different from initial J189 ICD-10 code assigned and agrees with DOHMH physician review of cause of death and ICD-10 coding			
Non-small cell lung cancer	C349 **(**Malignant neoplasm of bronchus or lung, unspecified)	Tobacco use (intermediate cause: lung cancer)	C349 (Malignant neoplasm of bronchus or lung, unspecified)^b^
Neutropenia	D70 (Neutropenia)	Wegener’s granulomatosis^c^ (intermediate cause: neutropenia due to medication)	D70 (Neutropenia)^b^
Anoxic brain injury	G931 (Anoxic brain damage, not elsewhere classified)	Anoxic brain injury following cardiopulmonary arrest	G931 (Anoxic brain damage, not elsewhere classified)
Amended underlying cause of death different from initial J189 ICD-10 but differs from DOHMH physician review of cause of death and ICD-10 coding			
*Providencia stuartii*	A414 (Sepsis due to anaerobes)	Dementia	F03 (Unspecified dementia)
Methicillin resistant s*taphylococcus aureus* urosepsis	N390 (Urinary tract infection, site not specified)	Status post cerebrovascular infarct due to cerebrovascular disease	I679 (Cerebrovascular disease, unspecified)
Intracranial hemorrhage nontraumatic	I629 (Nontraumatic intracranial hemorrhage, unspecified)	Accidental fall^d^	W19 (Unspecified fall)
*Stenotrophomonas maltophilia*	A498 (Other bacterial infections of unspecified site)	Breast cancer^c^	C509 (Malignant neoplasm of breast of unspecified site)
Bilateral pneumonia (*Klebsiella pneumoniae* and *Proteus mirabilis*)	B181 (Chronic viral hepatitis B without delta-agent)	Benign prostatic hypertrophy^c^	N40 (Hyperplasia of prostate)
*Klebsiella* urinary tract infection	N390 (Urinary tract infection, site not specified)	Vomiting due to ileus	K567 (Ileus, unspecified)
Hypertension	G20 (Parkinson disease)	Recurrent pneumonia^c^ (contributing cause: dementia)	F03 (Unspecified dementia)^b^
Pyelonephritis due to multi-drug resistant *klebsiella*	N12 (Tubulo-interstitial nephritis, not specified as acute or chronic)	Pyelonephritis (contributing cause: dementia)	F03 (Unspecified dementia)
Multiple cerebrovascular accidents – nontraumatic	I64 (Stroke, not specified as hemorrhage or infarction)	Multiple cerebrovascular infarcts	I639 (Cerebral infarction, unspecified)
Bladder cancer	C97 (Malignant neoplasms of independent [primary] multiple sites)	Tobacco use^c^ (Intermediate cause: Tracheostomy due to laryngeal and lung cancer)	C399 (Malignant neoplasm of lower respiratory tract, part unspecified)^b^
Chronic obstructive pulmonary disease	J440 (Chronic obstructive pulmonary disease with acute lower respiratory infection)	Cerebrovascular infarct	I639 (Cerebral infarction, unspecified)
Congestive heart failure	I500 (Congestive heart failure)	Asthma exacerbation	J459 (Asthma, unspecified)
Amendment does not identify condition that contributed to pneumonia or death due to pneumonia			
*Stenotrophomonas maltophilia* and *achromobacter* ventilator associated bilateral pneumonia	J159 (Unspecified bacterial pneumonia)	Subdural hematoma^d^	I620 (Subdural hemorrhage (acute, nontraumatic)
*Enterococcus faecalis*	J154 (Pneumonia due to other Streptococci)	Hypertension	I131 (Hypertensive heart and renal disease with renal failure)
*Pseudomonas aeruginosa*	J156 (Pneumonia due to other aerobic Gram-negative bacteria)	Dementia	F03 (Unspecified dementia)
*Candida albicans*	B371 (Pulmonary candidiasis)	Pulmonary fibrosis	J841 (Other interstitial pulmonary diseases with fibrosis)
*Candida albicans*	B371 (Pulmonary candidiasis)	Dementia	F03 (Unspecified dementia)
Methicillin resistant *staphylcoccus aureus*	J152 (Pneumonia due to *Staphylococcus*)	Dementia	F03 (Unspecified dementia)
*Pseudomonas aeruginosa*	J156 (Pneumonia due to other aerobic Gram-negative bacteria)	Anoxic brain injury^c^ (Contributing cause: *Clostridium difficile* colitis)	A047 (Enterocolitis due to *Clostridium difficile*)^b^
Beta-hemolytic *streptococcus* group 1	J154 (Pneumonia due to other Streptococci)	Atrial fibrillation not on anticoagulation	I48 (Atrial fibrillation and flutter)
*Proteus mirabilis*	J156 (Pneumonia due to other aerobic Gram-negative bacteria)	Right upper and right lower lobe pneumonia (Contributing cause: Dementia)	F03 (Unspecified dementia)^b^
Gram positive cocci in chains	J159 (Unspecified bacterial pneumonia)	Right lower lobe aspiration pneumonia (Contributing cause: Old cerebrovascular infarct)	I693 (Sequelae of cerebral infarction)^b^
Multilobar pneumonia, unspecified organism	J181 (Lobar pneumonia, unspecified organism)	Dementia	F03 (Unspecified dementia)
*Pseudomonas aeruginosa*	J156 (Pneumonia due to other aerobic Gram-negative bacteria)	Previous cerebral infarct	I693 (Sequelae of cerebral infarction)
Amendment did not change underlying cause of death or ICD-10 code			
Bilateral pneumonia	J189 (Pneumonia, unspecified organism)	Dementia	F03 (Unspecified dementia)
No amendment submitted (no response to query)			
Nothing submitted	Nothing submitted	Dementia	F03 (Unspecified dementia)

Abbreviations: ICD-10, International Classification of Diseases, 10^th^ Revision; New York City DOHMH, Department of Health and Mental Hygiene

a Literal (free-text) underlying cause of death and corresponding assigned ICD-10 code (9).

b In these cases, an intermediate or contributing cause was identified as the underlying cause of death by the standardized international algorithm.

c Review of case showed that the Office of the Medical Examiner should have been notified as per New York City guidelines (death involved therapeutic complication).

d Review of case showed that the Office of the Medical Examiner should have been notified as per national guidelines (death involved injury).

The DOHMH physician review found that the medical records related to all 29 queried deaths contained information about the cause of death that would have changed the underlying cause reported (Table). Underlying causes based on DOHMH review matched the underlying cause-of-death codes based on the clinician amendment in only 3 cases (10.7%). For 2 cases, no evidence was found that pneumonia was involved in the death at all. Additionally, the reviewing physician found that in 2 instances the certifying clinician should have reviewed the case with the medical examiner’s office because injury contributed to the death.

## Discussion

To our knowledge, this is the first study to document feasibility of querying cause of death, an NCHS-recommended best practice (6), using an electronic death registration system. Most health departments use time-consuming postal mail to query, requiring manual data entry of updated causes of death. In our study, electronic querying was operationally feasible, minimizing time between death and query initiation, reaching clinicians when they remembered case details better and while they were still at the reporting hospital, a key challenge in New York City where many are physicians-in-training who rotate to other locations. Nonetheless, query compliance and accuracy were major challenges.

Although the electronic messages emphasized that New York City’s health code required a prompt response, certifying clinicians had a low initial response rate. On follow-up, we found that nonclinical hospital staff members were unable to compel clinicians to respond. Therefore, we met with hospital medical and administration personnel, including medical department heads, to encourage their support. The administration expressed support for the querying system, agreeing to designate a clinician “champion” to facilitate timely query responses. Following this meeting, more queries were returned, but response quality, based on medical chart audit, was low. Amendments did not adequately address the underlying medical condition that put patients at risk for contracting or dying of pneumonia and were often inaccurate. Some inaccuracy probably occurred because the facility assigned a chief resident to respond to all queries instead of having the certifying clinician respond about his or her own certificates.

Our findings highlight the continued need to enforce New York City requirements for accurate cause-of-death information. We have disseminated new clinician education materials in the pilot hospital, updated a citywide online training module (12,13), and converted a public health epidemiologist’s position at DOHMH to a part-time physician position. The physician will conduct in-service trainings, follow up on unreturned queries, and audit medical charts to enable issuance of formal citations for inaccurate cause-of-death reporting. Given the limited time allocated to the task by a part-time position, the physician will focus on hospitals with large discrepancies between their billing discharge information and submitted cause of death based on assigned ICD-10 codes. We plan to expand this electronic death query system on a hospital-by-hospital basis with modifications to address noted challenges and will include a meeting with hospital administration prior to implementing the intervention to involve them in the effort.
